# Self correction of anterior crossbite: a case report

**DOI:** 10.4076/1757-1626-2-6967

**Published:** 2009-07-14

**Authors:** Chung Wai Mok, Ricky WK Wong

**Affiliations:** Orthodontics, Faculty of Dentistry, The University of Hong Kong2/F, 34 Hospital Road, Sai Ying Pun, Hong Kong SARPR China

## Abstract

A 9-year-old Chinese boy presented with an anterior crossbite, no treatment was performed at that time because the incisors have open root apices. The crossbite self-corrected after one year. This case demonstrated that an anterior crossbite may self-correct without treatment.

## Introduction

An anterior crossbite is the description of the upper anterior teeth having one or more occlusions at the lingual side of the lower anterior teeth. According to Lin JJ, the prevalence of anterior crossbite was 13.83% in a Taiwanese sample of 7090 elementary and junior high school students. Aged 9 to 15 years old [1]. The presence of anterior crossbites may cause mandibular displacement, if left untreated may lead to restriction of maxillary growth, traumatic occlusion, and may lengthen the treatment time.

## Case presentation

**Figure 1.**
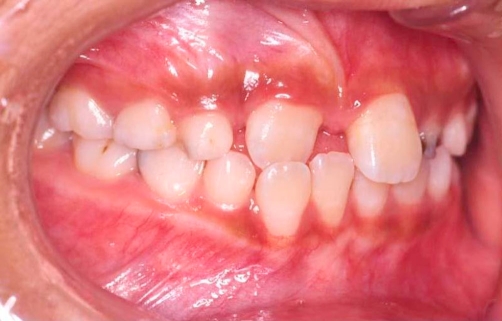
Tooth 11 in crossbite with tooth 42.

**Figure 2.**
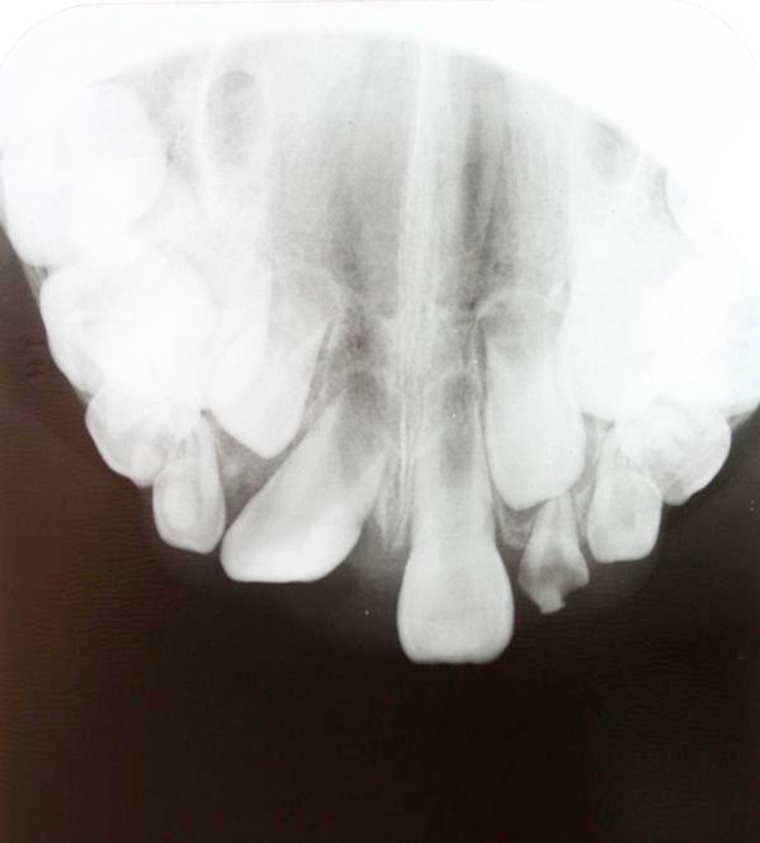
Apices of teeth 11 and 21 were still wide open.

**Figure 3.**
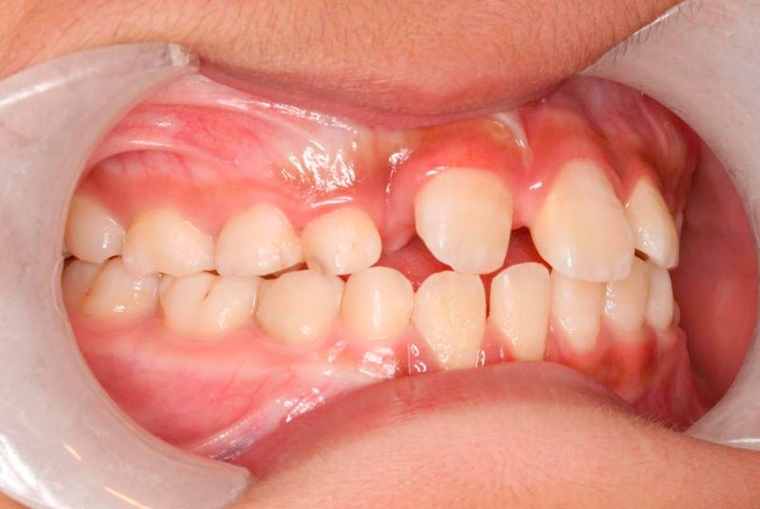
Tooth 11 in edge to edge position with tooth 42.

**Figure 4.**
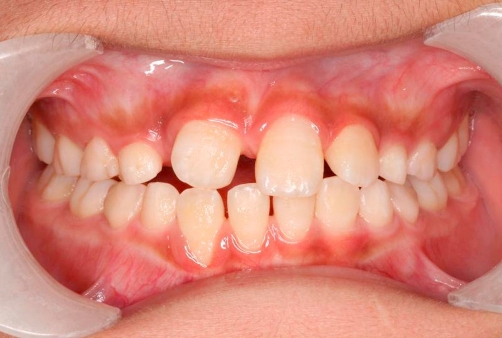
Buccal gingival recession of tooth 42 resulted from traumatic occlusion between teeth 11 and 42.

The patient was 9-year-old Chinese boy, was brought to our hospital by his mother to seek treatment for his “wrongly positioned front tooth”. His medical and dental histories were non-contributory. On extra-oral examination, his face was symmetrical with a slightly convex lateral profile. Intra-orally the tooth 11 was tipped disto-palatally and was in crossbite with the tooth 42 (Figure 1). Functional mandibular shift was not detected. Radiographic examination showed all permanent teeth developing normally. However teeth 11 and 21 still have wide open root apices (Figure 2). It was reported that orthodontic treatment of a tooth with an open root apex would produce early closure of the apex, resulting in a short-rooted tooth [2]. Therefore, it was decided to wait until the roots of the upper incisors were more developed before correcting the anterior crossbite. After one year the patient returned for review. The crossbite had improved and teeth 11 and 42 became edge to edge (Figure 3). However there was slight recession of tooth 42 possibly due to occlusal trauma (Figure 4). Since the crossbite was improving, it was decided to keep the case under observation.

**Figure 5.**
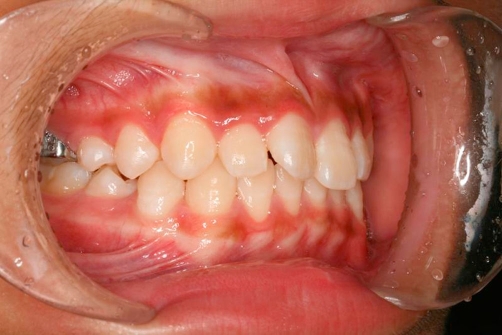
Anterior crossbite between teeth 11 and 42 corrected with normal overbite and overjet.

**Figure 6.**
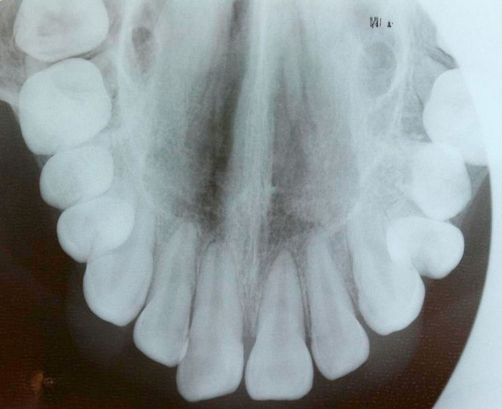
Root apex of tooth 11 matured with normal length.

The case was followed up to permanent dentition. A palatal arch type space maintainer was placed to minimise space loss in the upper arch. The crossbite further self-corrected to normal overbite and overjet. The gingival condition of 42 improved (Figure 5). Apices of teeth 11 and 21 were fully developed with normal root lengths (Figure 6). The band on tooth 16 (Figure 6) was the space maintainer.

## Discussion

An anterior crossbite may cause mandibular displacement which leads to various dental problems. Early correction of the anterior crossbite may facilitate the eruption of canines and premolars into Class I [3], eliminates traumatic occlusion to the incisors [4,5] (which may lead to dehiscence and gingival recession), providing a normal environment for growth of the maxilla [6], and can often improve the self esteem of the child [7-9]. Therefore early correction of the crossbite is indicated. In this case, correction of the anterior crossbite was postponed in view of the open apex. The crossbite subsequently self-corrected. It should be noted that this is not common. It is usually necessary to correct the crossbite by orthodontic means as an interceptive measure and this case was an exception to this general condition. It is possible that the tongue may have proclined the incisor, as there was space to allow this to occur.
